# Spontaneous Regression of Cerebral Arteriovenous Malformation Following Onyx Embolization

**DOI:** 10.7759/cureus.19533

**Published:** 2021-11-13

**Authors:** Eric Feldstein, Gerald Riccardello, Krishna Amuluru, Fawaz Al-Mufti, Chirag Gandhi

**Affiliations:** 1 Neurological Surgery, Westchester Medical Center, Valhalla, USA; 2 Radiology, Rutgers New Jersey Medical School, Newark, USA; 3 Radiology, St. Vincent Indianapolis, Indianapolis, USA; 4 Neurosurgery, Westchester Medical Center, Valhalla, USA

**Keywords:** interventional radiology guided embolization, cerebral avm, spontaneous regression, embolization, arteriovenous malformation

## Abstract

Little is known about the natural history of arteriovenous malformations (AVM) and less is known about their potential for spontaneous regression. The advent of endovascular treatment for embolization or pre-surgical embolization of cerebral arteriovenous malformations (cAVM) has seen several reports of spontaneous regression of partial embolization of cAVMs surface in the literature.

A 66-year-old patient had an initial diagnostic cerebral angiogram revealing a left frontoparietal region Spetzler-Martin (SM) grading 4 cAVM. The patient underwent three stages of embolization over eight months leading to a 90% reduction in nidal volume, before being lost to follow up for six years. A six-vessel diagnostic cerebral angiogram was performed at that time to assess for any interval changes and surprisingly, the previously visualized left frontoparietal AVM had regressed. There was evidence of prior onyx embolization with no residual filling or recurrence.

Spontaneous regression after partial embolization may be under-reported or the natural history is simply unable to temporally unfold because the typical treatment course results in surgery following partial embolization. Given the potential to forgo the risks of an open procedure, we believe this topic deserves further attention.

## Introduction

Cerebral arteriovenous malformations (cAVMs) are known to be complex tangles of abnormal arteries and veins associated with high-flow arteriovenous shunting, nidus formation, and venous ectasia. Little is known about their potential for spontaneous regression, a rare phenomenon with an estimated prevalence of 1-3% of patients with cAVM [1]. The majority of spontaneous regression cases in the literature occur either without treatment of any means or after partial surgery [2]. With the advent of endovascular treatment for complete embolization or pre-surgical embolization of cAVMs, several reports of spontaneous residual regression of partial embolization cAVMs have surfaced in the literature [3-6]. We report a case of complete regression of an unruptured AVM after partial endovascular embolization with Onyx, and review the literature of cases of spontaneous regression in partially embolized cAVMs in order to compare findings associated with each case.

## Case presentation

**Figure 1:**
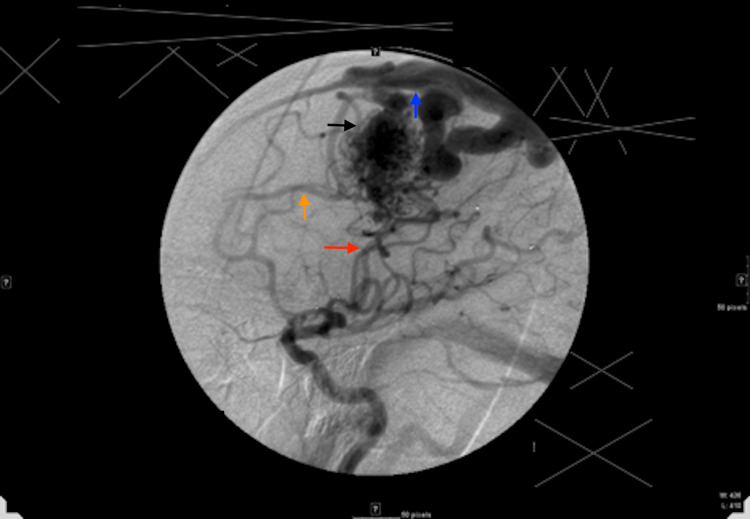
Initial diagnostic cerebral angiogram. Initial diagnostic cerebral angiogram revealing grade 4 left frontoparietal arteriovenous malformation (AVM) (black arrow) fed by the left anterior cerebral artery (orange arrow) and the superior division of the left middle cerebral artery (red arrow) and drained by superficial veins to the superior sagittal sinus (blue arrow).

**Figure 2:**
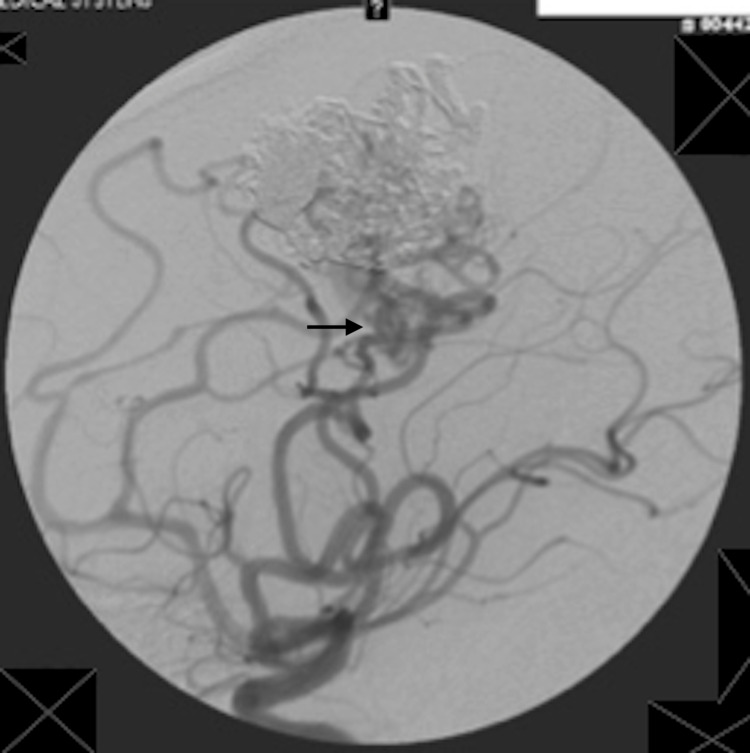
Post stage 3 embolization with Onyx with 85-90% reduction in AVM volume. AVM: Arteriovenous malformation.

The patient is a 66-year-old who had initially presented about eight years ago with the complaint of headache and left-sided tinnitus. The diagnostic cerebral angiogram revealed the left frontoparietal region (Spetzler-Martin [SM] grade 4 or embocure score of 5) cAVM which was fed by the left anterior cerebral artery (pericallosal and the callosomarginal) and the superior division of the left middle cerebral artery. The venous drainage was 2-3 mm deep to the superior sagittal sinus (Figure 1). The patient underwent stage I Onyx embolization with 30% reduction in the AVM nidus. About six months later, stage II Onyx embolization was performed further reducing the nidal volume by about 30% which was followed by stage III embolization after two months, during which the nidal volume was further reduced to a total of 90% (Figure 2).

**Figure 3:**
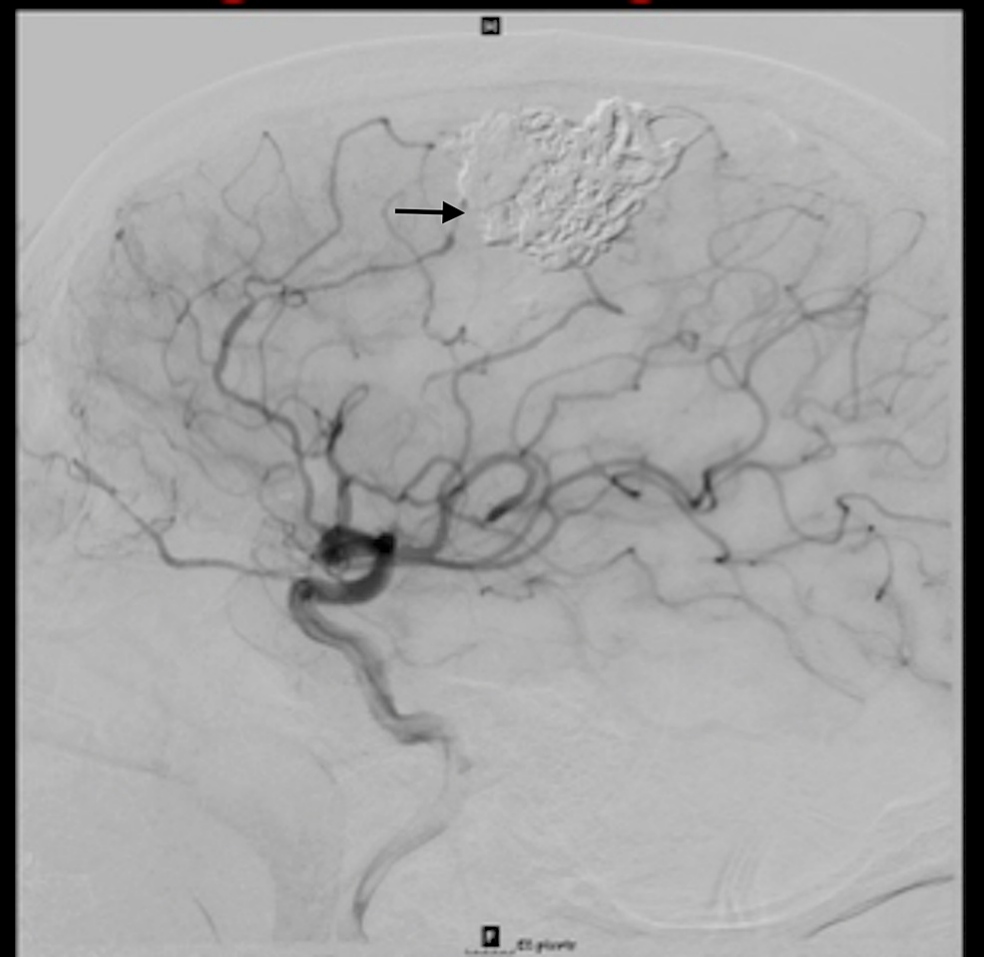
Follow-up six-years later revealing complete regression of AVM with evidence of prior Onyx embolization. AVM: Arteriovenous malformation.

The patient was lost to follow-up and presented again after about six years with the complaint of headache, dizziness, and tinnitus in the right ear. A six-vessel diagnostic cerebral angiogram was performed to assess for any interval changes and surprisingly, the previously visualized left frontoparietal AVM had regressed. There was evidence of prior Onyx embolization with no residual filling or recurrence (Figure 3).

Due to the patient's living situation and poor follow-up, it is hard to judge the long-term outcomes of their partial treatment with full regression. However, on their last admission, they had no evidence of complications due to their regressed cAVM.

## Discussion

Endovascular embolization therapy, which has been shown to be more efficacious and safer than surgery alone [7], is typically the first step of the multimodal approach to cAVM therapy with the goal of a permanent cure. Complete obliteration of cAVMs following endovascular therapy has been reported in up to 35% of cases [3], with the remaining partially embolized cases typically progressing to open or radiosurgery. In the case presented here, the patient underwent three embolization procedures with Onyx, with a final result of an 85-90% reduction of the unruptured SM grade 4 cAVM. We note that while the results of A Randomized Trial of Unruptured Brain AVMs (ARUBA) trial suggest that medical management alone is superior to interventional therapy for unruptured cAVMs, the mean follow-up of 33.3 months for this trial may not have been sufficient to capture the true risk comparison in these patients given the lifetime rupture risk of cAVMs [8]. As the patient was lost to follow-up, no additional surgeries or embolization were performed, yet the patient was found to have complete regression of the cAVM at six-year follow-up; thus, spontaneous regression of the remaining cAVM was suspected. 

A number of factors have been proposed for the spontaneous regression of cAVMs. The most suspected mechanism involves alterations in hemodynamics due to mass effects from intracerebral or subarachnoid hemorrhage brain edema and vasospasm leading to reduced blood flow and thrombosis [5]. AVM morphology is also suspected, with small size, single arterial feeding vessels, single ecstatic draining veins, and superficial locations appearing to play a causative role in spontaneous thrombosis [1,5]. In children and women taking oral contraceptives, it is believed a hypercoagulable state can predispose to the spontaneous thrombosis and regression of AVMs [9].

Spontaneous regression of whole untreated cAVMs is a rare phenomenon. While estimated to occur in as many as 20% of patients with cAVMs, most reports of spontaneous regression estimate a more conservative 1-3% [1,2,10]. Our own literature review of regression of partially treated cAVMs yielded only six other case reports (Table 1). The average age of these patients was 38 years old (ranging from 21 to 58). The cAVMs in five of the seven cases involved the parietal lobe, while the remaining two of seven involved the occipital lobe. A hematoma and vasogenic edema causing a slight mass effect was only found in one of the seven cases, while two of the seven cases presented with vasogenic edema without mass effect. A single feeding vessel was in zero of the seven cases, though a single venous draining vessel was found in four of the seven. Histoacryl ultrafluid Lipiodol ultrafluid mixture was used in four cases, Onyx in two, and N-butyl cyano-acrylate (NBCA) in one. Thrombosis or embolus was noted in the draining vein in three of the seven cases following embolization. Vasospasm, which is a previously noted potential factor in the spontaneous regression of cAVMs, due to catheter manipulation was noted in two of the seven cases. Time at the recognition of spontaneous regression in cases with regular follow-up was an average of 318 days, with a range of one day to 1186 days (six years in the current case, but the patient did not follow-up regularly). 

**Table 1:** Case series of seven spontaneously regressing cAVMs found in the literature. cAVM: cerebral arteriovenous malformations; S-M G: Spetzler-Martin grading; PCA: Posterior cerebral artery; AVM: Arteriovenous malformations.

Case	Reference	Age	Presentation	Location	S-M G	Feeding Vessels	Draining Vessels	Embolization	Angiographic Findings	Time to Regression
1	Cellerini M et al. (2003) [5]	48	Refractory seizures	Left parietal	3; 3 x 2 x 2 cm; Vasogenic edema surrounding nidus	Hypertrophic parietal branches of the left pericallosal and middle cerebral arteries	Three superficial veins coursing to the superior sagittal sinus and one deep vein running to the vein of Galen	20% occlusion via 0.4 and 0.2 cc of a 28% mixture of Histoarcyl and Ultrafluid (UF) Lipidiol	Thrombosis of the most posterior superficial draining vein	6 months
2	Cellerini M et al. (2003) [5]	28	Left hemifacial parasthesias extending to the fingers of the left hand and followed by central hemifacial palsy, dysarthria, and dysphagia	Left occipital pole	1; 2 x 2 x 1 cm central subcortical hematoma in the right cerebral hemisphere with vasogenic edema and slight mass effect	Superior and inferior branches off of rolandic branch of right middle cerebral artery	Single dilated vein of the head of the caudate nucleus coursing to the thalamostriate and internal cerebral veins	30% mixture of Histoacryl and Ultrafluid Lipiodol U.F; 2 via superior vessel, 1 via inferior to residual filling of nidus with sluggish flow of draining vein	Small embolus of glue passed into the draining vein, causing stagnant flow; vasospasm during catheterization	1 day
3	Cellerini M et al. (2003) [5]	54	Transient right hemianopia	2 x 2 x 1 cm; paraventricular white matter of the right parietal lobe	1; 2 x 2 x 2 cm; vasogenic edema surrounding nidus, without mass effect	Middle temporal branches and parietal occipital branches of the left middle and posterior cerebral arteries	Single cortical vein with stenosis at junction with the superior sagittal sinus	3 intranidal injections of a 20-50% mixture of Histoacryl and Ultrafluid into different feeding arteries	Spasm of PCA; Decreased ectasia and flow velocity of the vein to superior sagittal sinus; 80% reduction in nidus size	3 months
4	Cellerini M et al. (2003) [5]	26	Acute headache and vomiting	Left parietal lobe	1; 2 x 2 x 1 cm; no acute bleeding	Single dilated lenticulostriate artery and multiple small insular perforating branches of the right middle cerebral artery	Two deep veins, one of which was ectatic, coursing to the internal cerebral vein and straight sinus	3 attempts at insular perforating branch of the right middle cerebral artery were unsuccessful. A single injection of an 18% mixture of Histoacryl and Ultrafluid Lipiodol into the Lentriculostriate artery originating from the proximal M1 segment to the right middle cerebral artery	Glue reached the medial part of nidus; 50% occlusion of the nidus with a significant reduction in flow through the shunt and patency of the single draining vein	1 year
5	Cao C et al. (2015) [4]	31	Right hemiparesis	Right occipital cortical-subcortical region	2; no acute bleeding	Two main arterial feeders from the left pericallosal artery (superior and inferior parietal arteries) and tiny feeding branches from the left middle cerebral artery	Small cortical vein draining the AVM slowly into the superior sagittal sinus thrombosis of the AVM main draining vein	N-butyl cyano-acrylate (NBCA) with a 20% dilution into two feeding branches from the left pericallosal artery	Occlusion of the nidus with small peripheral capillary network surrounding; decrease in the size of the thrombosed vein at three months via MRI	39 months
6	Nas OF et al. (2017) [6]	21	Blurred vision, dizziness, nausea, headache	Left frontoparietal	2; 3 cm	P2 and P3 branches of the right posterior cerebral artery	Right sigmoid sinus through a single venous structure	Branches of the right pCA were selectively catheterized and Onyx injected	Residual AVM lesion could not be reached because feeding arteries were too thin	3 months
7	Current Case	58	Headache, tinnitus		4; no hemorrhage	Left anterior cerebral artery (pericallosal and the callosomarginal) and the superior division of the left middle cerebral artery	2-3 superficial veins to the superior sagittal sinus	Three stage embolization with Onyx: 6 and 8 months	85-90% reduction of volume size on final embolization	<6 years

## Conclusions

We were unable to find a single factor common to all cases of spontaneous regression following endovascular embolization; however, we note that all cases contain at least two of the factors hypothesized to contribute to spontaneous regression. We hypothesize that embolization agents result in progressive thrombosis due to reduced intranidal flow from both the embolization agent and catheter-induced vasospasm. Additionally, the continued thrombogenicity of the glue and the persistence of the inflammatory process following exposure of the vessels of the nidus to the embolic agent likely contribute to the spontaneous regression of partially embolized cAVMs as well. We hypothesize that spontaneous regression after partial embolization is either under-reported or the natural history is simply unable to temporally unfold because the typical treatment course results in surgery following partial embolization. However, given the risk of bleeding in partially embolized cAVMs and the inability to predict which cAVMs will spontaneously regress, it should not be considered as a treatment option. At this point, we cannot predict spontaneous regression after partial embolization and, given the potential to forgo the risks of an open procedure, we believe this topic deserves further attention.
